# Engineering At-Home Dilution and Filtration Methods to Enable Paper-Based Colorimetric Biosensing in Human Blood with Cell-Free Protein Synthesis

**DOI:** 10.3390/bios13010104

**Published:** 2023-01-06

**Authors:** Tyler J. Free, Ryan W. Tucker, Katelyn M. Simonson, Sydney A. Smith, Caleb M. Lindgren, William G. Pitt, Bradley C. Bundy

**Affiliations:** Department of Chemical Engineering, Brigham Young University, Provo, UT 84602, USA

**Keywords:** paper-based biosensor, cell-free protein synthesis, dilution, filtration, blood, colorimetric diagnostic

## Abstract

Diagnostic blood tests can guide the administration of healthcare to save and improve lives. Most clinical biosensing blood tests require a trained technician and specialized equipment to process samples and interpret results, which greatly limits test accessibility. Colorimetric paper-based diagnostics have an equipment-free readout, but raw blood obscures a colorimetric response which has motivated diverse efforts to develop blood sample processing techniques. This work uses inexpensive readily-available materials to engineer user-friendly dilution and filtration methods for blood sample collection and processing to enable a proof-of-concept colorimetric biosensor that is responsive to glutamine in 50 µL blood drop samples in less than 30 min. Paper-based user-friendly blood sample collection and processing combined with CFPS biosensing technology represents important progress towards the development of at-home biosensors that could be broadly applicable to personalized healthcare.

## 1. Introduction

Medical tests from patient samples provide critical information to guide the administration of healthcare to save and improve lives. Unfortunately, most clinical tests require a trained technician and specialized equipment. This limits most life-saving diagnostic information to affluent populations proximal to sophisticated laboratories [1,2]. Fortunately, some at-home tests have reached consumer markets for broad impact. Personal glucose monitors have empowered millions of diabetes patients to unlock better health outcomes through informed treatment management [3]. Other at-home tests for pregnancy, mold, toxic metals, glucose, and COVID-19 have the potential to inform personal and clinical decisions to promote better health outcomes [4,5,6,7]. Colorimetric at-home tests, such as the common over-the-counter pregnancy test, are particularly inexpensive and easy to use. However, such tests are not feasible with human blood due to its pigment. This work reports the effectiveness of inexpensive at-home-capable filtration and dilution methods which remove sufficient pigment to observe a colorimetric sensor response. This finding has the potential to enable the development of future at-home colorimetric diagnostics that detect important biomarkers in human blood.

There remains a vast unmet need of accessible blood diagnostics, particularly for routine testing which is important to the efficacy and safety of delicate cancer treatments [8,9,10,11,12,13,14]. Amino acid levels can be important clinical indicators to assess and guide cancer treatment [15,16,17]. Some chemotherapies directly target cancer cells with dysregulated amino acid metabolism [18,19]. “Glutamine addiction” is an Achilles heel of some cancer cells, and targeted intervention therapies show promising results [20,21,22]. CB-839, a chemotherapy that inhibits glutaminase, interferes with glutamine metabolism which initiates toxicity toward cancer cells, but at some concentrations it is also toxic to healthy cells, causing dangerous side effects [18,23]. Glutamine monitoring has been included as a part of clinical trials (NCT03872427 and NCT04634539) to characterize the expected correlation between glutamine levels and cancer patient outcomes. Personalized glutamine monitoring could be a valuable tool to enable patient-specific optimization of cancer treatment to improve patient outcomes [24,25]. Glutamine has also been reported as a clinically relevant biomarker for various other diseases, including kidney disease, and diabetes [26,27,28,29,30]. Unfortunately, glutamine tests are typically conducted by a trained technician in a lab with specialized equipment, which severely limits or prevents routine monitoring [31,32,33,34].

To directly address the unmet need for accessible glutamine monitoring tests, a low-cost cell-free protein synthesis (CFPS) glutamine assay was recently developed [24]. CFPS utilizes an accessible and tunable reaction environment to make specialized protein products including magistral therapeutics, virus-like particles, biosensors, and gene circuits [35,36,37,38]. CFPS technology has recently been engineered to make low-cost (<$1 for materials), rapid (<60 min), scalable, equipment-free diagnostics used in proof-of-concept studies to detect a variety of clinically relevant compounds, including pathogens, toxic compounds, and amino acids [38,39,40,41,42]. CFPS systems can be lyophilized for shelf stability in a paper-based colorimetric embodiment to enable equipment-free on-demand sensing with a variety of sample types [38,39,40].

Physiological processes keep many blood substrate levels within a relatively stable range [43,44]. Deviations from normal ranges are often associated with disease, making blood diagnostics clinically useful to assist in disease diagnosis and management [45,46]. CFPS proof-of-concept studies report the biosensing of clinically relevant molecules in blood and serum, though optimization and clinical validation must precede widespread clinical use [24,39,40,47]. Early stages of field validation have already begun for a CFPS zika virus biosensor [48]. Low-cost shelf-stable CFPS biosensors with optical signals are appealing for at-home routine analysis, but blood turbidity interferes with the ability to detect a colorimetric response. This work demonstrates dilution and filtration as two inexpensive equipment-free methods to enable a colorimetric CFPS reaction. As a proof-of-concept, the dilution method is used to reduce the optical interference of blood to enable a colorimetric biosensor responsive to glutamine in 50 µL blood drop samples.

Red blood cells are about 8 µm in diameter and comprise approximately 35 to 47% of the total volume of blood which results in unique microfluidic behavior [49,50,51]. The cells are largely responsible for the unique non-Newtonian microfluidic behavior of blood as well as the optical interference [50,51]. Academic and industrial research teams have engineered different ways to separate red blood cells from serum to make diagnostic devices compatible with whole blood [52,53,54,55]. Centrifugation is the gold standard lab-based method to separate blood cells from serum. Hand-held centrifugal devices have been wisely suggested for point-of-care sample processing to extract serum [40,56,57], but this sample processing adds an extra step and possible variation to point-of-care sample collection. This work demonstrates the potential of two alternative methods for blood sample processing that can enable simple protocols for an at-home user. Using simple materials, dilution and filtration are presented as viable at-home sample processing methods to minimize the optical interference of red blood cells to enable clear serum extraction.

Filtration and other types of microfluidic separation are promising techniques for enabling direct passive addition of serum to embedded diagnostic reagents [58,59]. Filtration membranes for this application are often expensive as they must be carefully designed to extract serum without hemolysis or flow obstruction [52,60,61,62,63,64,65,66,67,68,69]. Additives such as agglutination agents have been added to porous filters to aggregate red blood cells, enabling serum extraction with a wider range of pore sizes which could reduce the cost of this approach [70,71,72,73]. Microfluidic devices with precisely sized channels have also successfully separated red blood cells for serum diagnostics [74,75,76,77,78]. Microfluidic devices and filtration membranes often require sophisticated manufacturing which often incurs a burdensome cost which limits the widespread distribution of diagnostics [70,77].

Blood dilution has been previously reported as a simple way to enable CFPS diagnostic compatibility [39]. Dilutions are commonplace in laboratories, but accurate dilutions can be difficult to obtain at home without laboratory equipment. To enable an accurate blood dilution, a precise volume of a blood sample is mixed with a precise volume of diluent. A premeasured amount of diluent can be shipped to the home of a patient, but a precise volume of blood is more difficult for a patient to collect at home. While lancets used to draw a finger prick blood sample are a viable at-home blood collection method, the collected volume varies considerably [79], which compromises the accuracy of a dilution and therefore, compromises the accuracy of test results. To address this need, this work utilizes commercially available MICROSAFE^®^ pipettes to collect a precise blood volume from a finger prick to enable at-home dilutions of blood for use in a proof-of-concept glutamine sensor. The capillary blood collection devices have impressive accuracy reports in previous studies [80,81].

**Figure 1:**
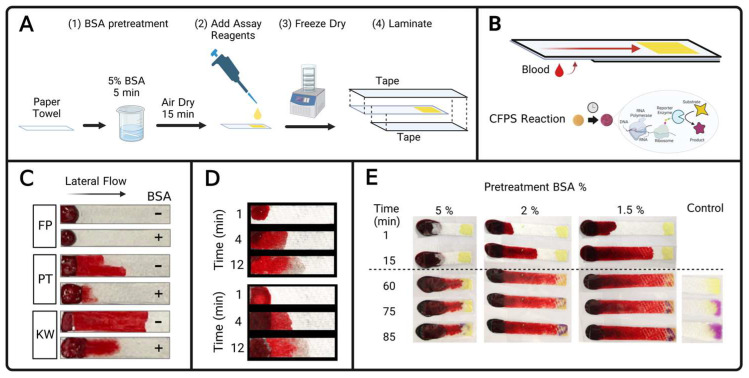
Low-cost serum extraction from blood for cell-free protein synthesis (CFPS) colorimetric reactions. (**A**) Assembly workflow of a prototype filtration test. Paper is first soaked in a 5% BSA solution and allowed to air dry. Assay reagents are pipetted onto the papers which are then air dried or freeze dried. Papers are then laminated with tape on both sides, except for a sample entry area which is not taped. (**B**) Assembled diagnostic in which human blood is applied to the underside sample entry area and capillary action induces lateral flow of the sample to the reagents. Hydration initiates a colorimetric CFPS reaction which produces an enzyme, β-galactosidase, which converts the yellow substrate chlorophenol red β-d galactopyranoside (CRPG) into a purple product. (**C**) The blood filtration capability of different materials with (+) and without (−) BSA blocking. FP is filter paper, PT is paper towel, KW is Kim wipe. Photographs were obtained after 15 min of lateral flow filtration had occurred. (**D**) Two replicates of a BSA blocked and tape-laminated paper towel in a blood filtration time course, with photographs from duplicate experiments shown after 1, 4 and 12 min. (**E**) Photographs of generic colorimetric CFPS reactions hydrated with serum that is passively extracted from blood via filtration by a BSA-blocked paper towel. 60 μL of fresh blood was promptly pipetted onto a flat surface and a pre-made filtration lateral strip was placed so that the exposed area of the paper was placed on top of the blood drop. Each column of photographs represents a series of photographs of the same lateral strip, at the times indicated on the vertical axis. The dashed line marks that filtration occurred at room temperature, but after 60 min the test strips were placed on a 40 °C hot plate for the cell-free reaction. The horizontal axis denotes the composition of the BSA solution that the paper strip was soaked in prior to drying. The right-most column is a paper strip that was hydrated with water instead of blood, and immediately placed on the hot plate at the same time as the other reactions.

**Figure 2:**
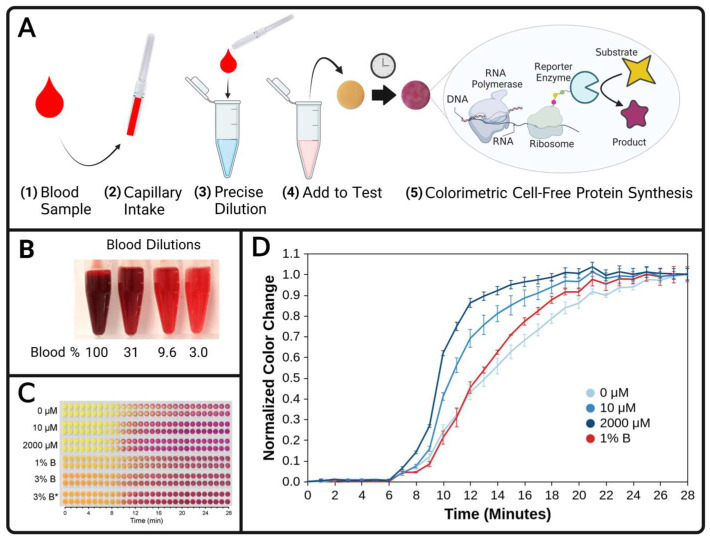
A method of at-home blood dilution compatible with CFPS biosensing reactions. (**A**) Workflow of a CFPS blood dilution diagnostic. Blood is collected, diluted, and then used to hydrate a prepared CFPS reaction wherein the coupled transcription and translation of the β-galactosidase reporter enzyme converts the yellow substrate, chlorophenol red β-d galactopyranoside (CRPG), to the purple product chlorophenol red. (**B**) Serial 3.22-fold dilutions of human blood from left to right. The percentages indicate the blood concentration (% *v/v*) in deionized water. (**C**) Reaction time-course photographs of a glutamine biosensor and various samples and standard reactions. Glutamine-deficient CFPS reagents lyophilized on paper are hydrated with 12 µL of fluid. The top 3 labels with concentrations, in µM, indicate the glutamine concentrations in aqueous standards. The bottom 3 labels with percentages indicate a blood volume ratio, diluted in water. The 3% B* indicates a 3% blood dilution sample that was spiked with 2000 µM of added glutamine. Each condition was performed in duplicate and the photographs of duplicates were arranged in adjacent rows, where each row represents repeated photographs of the same reaction paper. (**D**) Quantitative analysis of sensing reaction photographs for selected conditions from C. The average color for each circular reaction paper at each time point was quantified in CIELAB color space and normalized to the starting and ending values within each row. For each time point, the average of replicates for each condition was plotted with interpolating lines. The standard deviation for each set of duplicate reactions is displayed as error bars. A plot with the individual data points can be found in Supplementary Figure S6 and a plot including quantification for 3% B and 3% B* is included in Supplementary Figure S1.

Despite vast research efforts to develop blood separation techniques for diagnostic assays, cheaper and easier sample collection and processing methods are needed to interface with at-home blood biosensors. To this end, this work demonstrates specific filtration and dilution methods as two low-cost methods to sufficiently reduce the optical interference of blood resulting in visually unambiguous CFPS colorimetric reactions (illustrated in Figure 1A,B and Figure 2A).

## 2. Materials and Methods

### 2.1. Human Blood Samples

After obtaining institutional IRB approval, human blood samples were obtained from consenting volunteers in accordance with approved safety guidelines. Whole blood was collected by venipuncture into vacutainer tubes containing sodium heparin, EDTA, or no additives. Preliminary materials screening experiments were conducted with EDTA stabilized blood. Filtration experiments with colorimetric cell-free protein synthesis utilized blood with no additives, and dilution experiments were conducted with heparinized blood.

### 2.2. Blood Filtration

Filter paper (VWR quantitative 474 filter paper, VWR International, Radnor, PA, USA), Kim wipes (Kimberly-Clark Kimtech Kimwipes delicate task wipers, Roswell, GA, USA), and paper towels (Item 89460, Georgia-Pacific, Atlanta, GA, USA) were selected for preliminary screening tests. These prospective filtration materials were prepared with and without Bovine Serum Albumin (BSA) in anticipation of possible hemolysis due to surface interactions with erythrocytes. Filter materials were soaked in 5% BSA and allowed to dry at 37 °C for 15 min.

Human blood was added to filtration materials in drops of 20 to 100 µL and papers were monitored for preferential separation of clear liquid in flow spreading laterally or through the paper. Photographs were subsequently collected for documentation using an iPhone or canon camera.

For lateral flow filtration, filtration materials were cut to a width of approximately 10 mm and variable lengths, most commonly 30 mm. For lateral strips with cell-free reactions, reagents were pipetted onto paper and dried. Transparent tape (JVCC BOOK-20CC, J.V. Converting Company, Inc., Fairless Fields, PA, USA) was used on both sides of the lateral flow filter to create a sealed chamber for liquid to travel in lateral directions.

### 2.3. Blood Dilution and Pipetting

All pipetting for this work was performed with polypropylene pipette tips used with Rainin^®^ LTS pipettors (Pipette.com, San Diego, CA, USA) except the experiments shown in Figure 2C and Figure S2 which utilized disposable pipettes. Where used, field-ready disposable 50 μL MICROSAFE^®^ pipettes (SAFE-TEC, Broomall, PA, USA) were used according to the manufacturer’s instructions to collect blood (Step 2 of Figure 2A) and immediately dispense it into a prescribed volume of diluent (Step 3 of Figure 2A which was used for the dilutions used in Figure 2C) or onto a scale (Ohaus, Parsippany, NJ, USA) for validation experiments (Figure S2).

### 2.4. Cell-Free Protein Synthesis

Chemical reagents were purchased from Cayman Chemical (Ann Arbor, MI, USA) unless otherwise noted. *E. coli* cell extract served as the source of transcription and translation catalysts for cell-free protein synthesis reactions. The extract was prepared according to previously published protocols [82,83]. Briefly, BL21-Star™ (DE3) ΔLac cells were grown in autoclaved 2xYT media at 37 °C and 280 RPM. First, cells were grown in 5 mL of media overnight, and transferred to 100 mL of fresh media. When the cells reached an OD600 of 1, the volume was transferred to 900 mL of fresh media. When OD600 reached 0.5, production of T7 RNA polymerase was induced by the addition of isopropyl β-d-1-thiogalactopyranoside (IPTG) to a final concentration of 1 mM. Cells were allowed to grow until OD600 was between 2 and 4. Cells were harvested by centrifugation at 6000 RCF for 15 min and stored at −80 °C. Cells were resuspended in 10 mL per gram of wet cells in buffer A (14 mM magnesium acetate, 10 mM tris, 60 mM potassium glutamate, and 1 mM dithiothreitol) and centrifuged again at 6000 RCF for 15 min. The supernatant was discarded and the cells were resuspended in 1 mL buffer A per gram of cells. The resulting cell suspension was lysed by 3 passes through a French press homogenizer (Avestin, Ottawa, CA, USA). Cell lysate was clarified by centrifugation at 12,000 RCF for 30 min. The supernatant was recovered, incubated for 30 min at 37 °C with 280 RPM shaking, then aliquoted and stored at −80 °C.

To energize the *E. coli* lysate, the PANOxSP reagents were prepared according to previously published protocols aimed at improving protein synthesis [24,84]. The cell-free reactions contained 18 mM magnesium glutamate, 1 mM 1,4-diaminobutane, 1.5 mM spermidine, 40 mM phosphoenolpyruvate, 10 mM ammonium glutamate, 175 mM potassium glutamate, 2.7 mM potassium oxalate, 0.33 mM nicotinamide adenine dinucleotide, 0.27 mM coenzyme A, 1.2 mM ATP, 0.86 mM CTP, 0.86 mM GTP, 0.86 mM UTP, 0.17 mM folinic acid, and 2 mM of the proteinogenic amino acids except glutamate. For the glutamine assay reactions, glutamine was omitted from the PANOxSP preparation and 80 mM methionine sulfoximine (MSO) was added to inhibit endogenous glutamine synthetase [24].

Protein synthesis of the β-galactosidase reporter enzyme resulted in the conversion of chlorophenol red-β-d-galactopyranoside (CRPG) (Gold Bio, St Louis, MO, USA) from yellow to purple. Cell-free reactions contained 12–24 nM LacZ plasmid, 0.6 mg/mL CRPG, 25% (*v/v*) cell extract, and the balance of the reaction volume was water. RNase inhibitor was expressed in separate cell-free reactions and added to the master mix at 3% (*v/v*) [85]. For tests anticipating diluted blood, 12 µL of CFPS reagents was pipetted onto hole-punched chromatography paper (Thick Chromatography Paper Grade 238, VWR 28342−036, VWR International, Radnor, PA, USA) circles and lyophilized (FreeZone 2.5, Labconco Corporation, Kansas City, MO, USA) [82]. For reactions anticipating filtrate from pure blood, the master mix was directly pipetted onto the BSA-blocked paper towel and air dried at room temperature. Air-dried reagents were stored at −20 °C until use.

### 2.5. Glutamine CFPS Biosensing

The CFPS glutamine sensing reaction papers were placed on a plastic plate at 37 °C and hydrated simultaneously with 12 µL of liquid samples. The samples were immediately covered with transparent tape (JVCC BOOK-20CC, J.V. Converting Company, Inc., Fairless Fields, PA, USA) to minimize evaporation. A canon camera captured images containing all test papers at t = 0, 1, 7, and every subsequent minute thereafter until t = 28 min. The image at t = 1 was duplicated 6 times to interpolate during the period of 2 to 6 min, which was prior to the observed color change. Each image was first cropped into narrow rectangles containing the circular test papers and aligned to show the sequential appearance of each test for the duration of 28 min. Next, an HSV saturation threshold identified the circles in the image based on their contrast with white background. The RGB values for all pixels in each circle were then averaged to obtain a representative color for each circle. The representative colors were then converted from RGB to CIELAB color space and the difference from the initial time point was computed using Euclidean distance. The averaged color of the first and last photo within each row were used as the low and high values, respectively, by which to normalize the data within each row.

## 3. Results and Discussion

### 3.1. Blood Filtration with Paper and CFPS Compatibility

Materials with different microfluidic properties were tested with the end goal of finding a less expensive material than commercial blood filter membranes [53,62,86,87,88]. Figure 1C reports the filtration performance of three types of inexpensive and readily-accessible paper types: filter paper (VWR quantitative 474 filter paper, VWR International, Radnor, PA), Kim wipes (Kimberly-Clark Kimtech Kimwipes delicate task wipers, Roswell, GA, USA), and paper towels (Item 89460, Georgia-Pacific, Atlanta, GA, USA), with and without BSA pre-treatment. The microstructure of some materials, including paper, can rupture red blood cells [89]. BSA blocking was hypothesized to coat the material surface with protein to reduce hemolysis derived from the microstructure of paper [90]. Figure 1C illustrates the transport of an opaque red fluid along the length of the wetting distance for materials not treated with BSA. Within a BSA-pretreated paper towel (Figure 1D), clear liquid travels ahead of the red pigment, indicating successful separation of red blood cells.

After BSA-treated paper towels were found to be a suitable material for clear serum extraction, compatibility with CFPS reagents was verified. CFPS reagents primed to make β-galactosidase for a color-change reaction were pipetted and dried directly on the end of BSA-treated paper towel to enable one-step filtration wherein the transparent filtrate passively hydrates test reagents to initiate a colorimetric reaction (Figure 1E). These results confirm that CFPS can be successfully utilized in conjunction with lateral flow blood filtration with a BSA-blocked paper towel (Figure 1E), which is a necessary finding for future work to use this technology in a CFPS biosensor.

While these low-cost filtration results are encouraging, several engineering challenges remain. BSA-treated paper towels consistently provide qualitative extraction of clear serum from raw blood samples, but the filtration speed and linear distance of lateral flow were observed to vary substantially between experiments. Sample-to-sample variation (e.g., hematocrit) is likely a contributing factor as is the extent of BSA pretreatment of the paper towel, which has yet to be thoroughly optimized. An increased extent of BSA pretreatment decreases the capillary action that causes wicking flow, which motivated the fabrication of shorter paper strips for increased BSA as shown in Figure 1E. The dried CFPS reagents must be located sufficiently far from the starting blood sample to ensure that red blood cells do not reach them, but sufficiently close that the clear serum can reach the reagents in a timely manner. The BSA paper towel filtration method exhibits exciting potential for low-cost blood separation and these issues, while formidable, can likely be improved in future work with careful design and optimization.

### 3.2. Blood Dilution Enables Paper-Based Colorimetric Sensing of Glutamine with CFPS

Dilution is the second low-cost method assessed in this work for sufficient reduction of blood turbidity to enable a visual colorimetric CFPS readout. As detailed below and illustrated in Figure 2A this approach will be applied to glutamine sensing in human blood (Figure 2A). However, initial dilutions of human blood in microcentrifuge tubes demonstrated notable turbidity even when diluted to 3.0% *v/v* (Figure 2B). Fortunately, when microliters of blood diluted to 3% are applied to filter paper, only a slight change of the color appears (compare pictures of the top six circular filter paper tests in Figure 2C at time 0 with the bottom six tests in Figure 2C which contain 12 µL of blood prediluted to 1% and 3%).

To engineer a colorimetric biosensor responsive to glutamine from a sample, glutamine-deficient CFPS reagents [24] were combined with a sample to enable production of a reporter enzyme, β-galactosidase, at a rate proportional to the sample glutamine level. The colorimetric substrate, chlorophenol red β-D galactopyranoside (CRPG), is yellow but the reporter enzyme catalyzes a reaction yielding a purple product (pictures of yellow and purple circles shown in Figure 2A). Thus, protein synthesis rate comparisons, and relative glutamine concentrations, can be correlated from visual results [38]. Reagents were pipetted onto chromatography paper discs and lyophilized (CFPS reagents are shelf-stable for months after lyophilization) [82]. Control tests were performed where water samples with 0 µM, 10 µM and 2000 µM of glutamine were added to these paper discs and incubated. As shown in the top two tests in Figure 2C, there is some background production of the β-galactosidase enzyme. As previously reported, methionine sulfoximine (MSO) was added to inhibit glutamine synthetase [24], but MSO does not enable complete suppression, as indicated by the gradual color change of the 0 µM reactions (Figure 2C). Prior to commercialization, the time-dependence of the assay readout and dynamic range need to be improved and rigorously characterized but, the primary purpose of demonstrating the glutamine sensor featured in this work is to report that diluting human blood enables a paper-based colorimetric biosensor readout.

To obtain a semiquantitative colorimetric test result, an observer can rank the color of an unknown sample with the colors observed in standard reactions to rank an unknown concentration. Analyte concentrations of standard reactions can be strategically selected to benchmark clinically relevant concentrations for a desired sensing application. The blood concentration of glutamine for a healthy human is between approximately 400 and 900 µM [91], and clinically relevant levels span 200 to 1400 µM [24]. The glutamine assay presented in this work has a sensitivity range that can discern between 0 and 10 µM glutamine, however, a visually obvious change was not observed between the 10 and 2000 µM glutamine control samples (Figure 2C). Thus, without dilution, clinically relevant blood glutamine levels would likely saturate this assay.

The colorimetric glutamine sensor reported herein is fortuitously suited for diluted blood which (1) reduces glutamine levels into the sensitivity range of the assay and (2) reduces the blood turbidity to enable the observation of colorimetric test results. Indeed when 12 µL of 1% or 3% diluted blood sample was added to the paper, the slight pink hue did not obscure the CFPS color change (Figure 2C bottom six tests). If the blood donor for the experiment shown in Figure 2C had glutamine levels in the physiological range, a 3% solution would contain between 12 and 27 µM, and a 1% solution would contain between 4 and 9 µM. The color change of the 1% blood sample is faster than the 0 µM control but slower than the 10 µM control, as seen by the color difference which is observable for a few minutes at about time = 15 min. Photographs of the test papers are shown in Figure 2C and analyzed quantitatively in Figure 2D. Thus, the semiquantitative test result from this proof-of-concept study suggests that the original blood sample contained greater than 0 µM glutamine but less than 10 µM. Additional aqueous control reactions [40] with clinically meaningful glutamine concentrations, such as 4 µM and 9 µM to span the clinically relevant range of 1% blood, could enable semiquantitative test results to indicate anomalous glutamine levels. Additionally, as a control for possible CFPS inhibition by blood components, one aliquot of blood was spiked with concentrated glutamine in a negligible volume raising the sample glutamine concentration by 2000 µM, and the color change rate with the excess glutamine appears to show little or no reaction hinderance by blood (3% B* in Figure 2C, and Supplemental Figure S1).

Finally, to enable at-home tests with small samples of diluted blood, sample collection devices must provide accurate dilutions without an equipment requirement. Accurate dilutions are readily performed in a laboratory, as calibrated micropipettes can reproducibly provide precise volumes at the microliter scale. To enable at-home sample collection, MICROSAFE^®^ disposable pipettes were used in this study to obtain 50 µL quantities of blood to perform blood dilutions for glutamine testing (Figure 2C). The devices do not require a technician or expensive equipment and are thus ideal for use at home. Validation experiments in this work using human blood demonstrated that MICROSAFE^®^ 50 µL pipettes have accuracy and precision that is generally comparable to Rainin^®^ LTS pipettes with polypropylene tips for human blood samples (Supplemental Figure S2). The disposable pipettes can be designed for one of various fixed volumes to accurately transfer blood volumes as small as 5 μL and as large as 80 μL [80,81], which are all within the collection range of finger prick volumes of blood and the volumes needed for this assay. A wide range of possible blood pipetting volumes enables a wide range of dilution ratios, which can be further expanded by varying the premeasured diluent volume.

## 4. Conclusions

The increase in demand for at-home diagnostic tools has driven scientific advancements over the past decades. Colorimetric diagnostics have a convenient equipment-free readout, but biosensing in raw blood obscures the visible response. This work demonstrates two methods, filtration and dilution, to overcome the optical interference of blood to advance biosensing technology towards low-cost at-home diagnostics. The former method utilizes BSA-blocked paper towels as a low-cost potential alternative to expensive membranes for passive serum extraction from blood, but further engineering is needed to enable dependable blood biosensing. The latter method utilizes disposable pipettes to collect a precise volume from a small drop of blood to mix with a premeasured volume of diluent water to enable the end user to perform a reliable dilution without technical training or expensive equipment. An amount of 50 µL of human blood is diluted using this method for a colorimetric paper-based glutamine biosensor with a time-dependent readout in less than 30 min. These two methods to reduce blood turbidity in low-resource settings could have broad applicability for future efforts to engineer other biosensing technologies to interface with human blood samples. This work contributes progress toward the development of at-home diagnostics that can potentially provide individuals with personalized medical information to inform personal healthcare decisions, potentially improving patient prognosis and quality of life.

## Data Availability

Not applicable.

## Supplementary Materials

The following supporting information and source code for image colorimetric quantification can be downloaded at: https://www.mdpi.com/article/10.3390/bios13010104/s1; Figure S1: Additional image color quantification; Figure S2: Pipette comparison; Figure S3: Original photographs used for Figure 1D; Figure S4: Original photographs used for Figure 1E; Figure S5: Standard Deviation Equation; Figure S6: Glutamine sensor response plot including individual data points; and Table S1: Cost estimate for one replicate of a CFPS blood filtration test; and Python Source Code to quantify Figure 2C color differences.py: source code for image color quantification. References [92,93] are cited in the supplementary materials.
