# Supplementary material for: Engineering At-Home Dilution and Filtration Methods to Enable Paper-Based Colorimetric Biosensing in Human Blood with Cell-Free Protein Synthesis
Source: Biosensors (Basel). 2023 Jan 6;13(1):104. doi: 10.3390/bios13010104 (PMC9855769; doi:10.3390/bios13010104)
Supplement: Supplementary file 1 [file biosensors-13-00104-s001.zip › biosensors-2102695-supplementary .pdf]

biosensors

MDPI

Article

Supplementary Materials

Engineering At-home Dilution and Filtration Methods to Enable Paper-based Colorimetric Biosensing in Human Blood with Cell-free Protein Synthesis

Tyler J. Free[1 †], Ryan W. Tucker[1 †], Katelyn M. Simonson[1], Sydney A. Smith[1], Caleb M. Lindgren[1], William G. Pitt[1], Bradley C. Bundy[1*]

[1] Department of Chemical Engineering, Brigham Young University, Provo, UT 84602
* Correspondence: B.C. Bundy, email: bundy@byu.edu
† These authors contributed equally to this work

Publisher's Note: MDPI stays neutral with regard to jurisdictional claims in published maps and institutional affiliations.

Copyright: © 2023 by the authors. Submitted for possible open access publication under the terms and conditions of the Creative Commons Attribution (CC BY) license (https://creativecommons.org/licenses/by/4.0/).

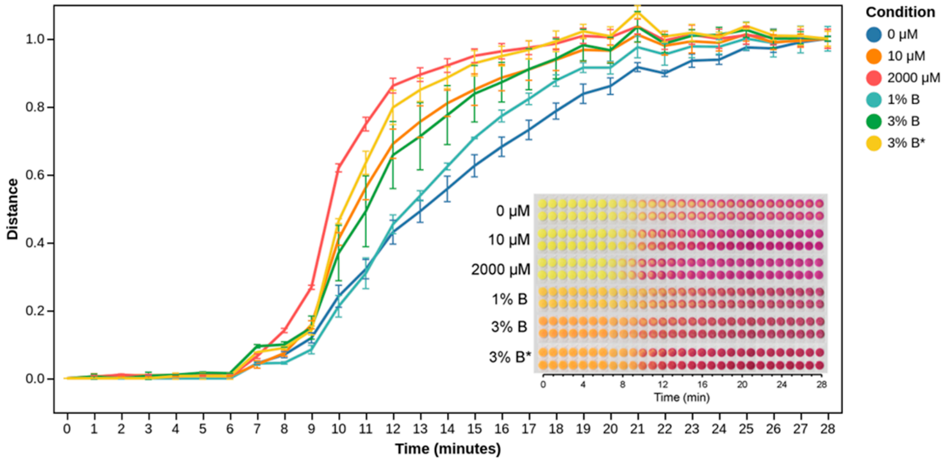

Supplemental Figure S1: Additional image color quantification. Quantification of each image included in Figure 2C with error bars representing the standard deviation for n = 2 paper tests. The vertical axis represents the normalized distance in CIELAB color space of each image relative to the initial and final images in the time course. Each image was first cropped into narrow rectangles containing the circular test papers and aligned to show the sequential appearance of each test for the duration of 28 minutes. Next, an HSV saturation threshold identified the circles in the image based on their contrast with white background. The RGB values for all pixels in each circle were then averaged to obtain a representative color for each circle. The representative colors were then converted from RGB to CIELAB color space and the difference from the initial time point was computed using Euclidean distance. The averaged color of the first and last photo within each row were used as the low and high values, respectively, by which to normalize the data within each row. 1% B indicates that the paper disc was hydrated with a solution of blood diluted 100-fold in water, with a final concentration of 1 % (v/v). Similarly, 3% B corresponds to a blood concentration of 3 % (v/v). 3% B* indicates a reaction hydrated with a glutamine-spiked blood aliquot. A negligible volume of concentrated glutamine was added to a blood aliquot to add 2 mM glutamine beyond the sample's original contents.

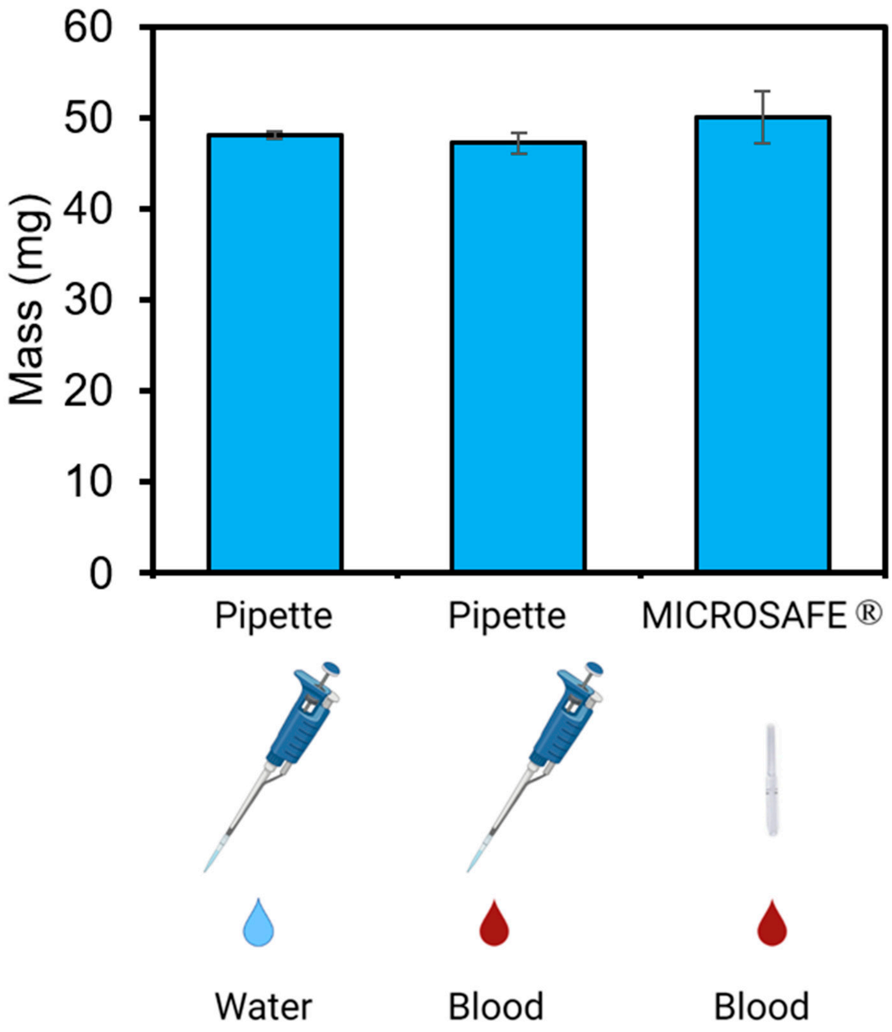

Supplemental Figure S2: Pipette comparison. Mass measurements of liquid dispensed by MICROSAFE® pipettes and conventional micropipettes. Conventional micropipettes (RAININ LTS) with fresh polypropylene tips were adjusted to dispense 50 μL of either water or blood. MICROSAFE® disposable pipettes are available in various sizes, and a 50 μL size was used for this study. The dispensed liquid, either blood or water, was aspirated and dispensed on a precision scale (OHAUS) and the mass was recorded. Error bars indicate the standard deviation for n = 6 measurements. For reference, the specific gravity of blood has been reported to be approximately 1.04 to 1.06 [1].

A B C

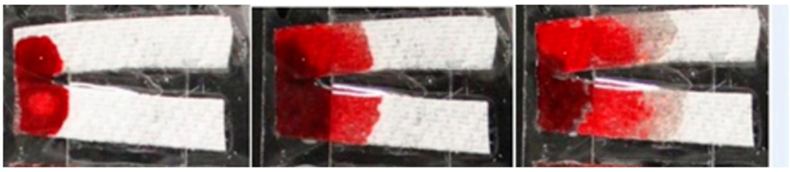

Supplemental Figure S3: Original photographs used for Figure 1D. Sequential photographs of filtration of whole blood with paper towel that was pretreated with 5% BSA. BSA-blocked paper strips were prepared as outlined in Figure 1A, with the omission of the CFPS reagents, and subjected to fresh blood for lateral flow filtration. A) Image after 1 minute of filtration. B) Image after 4 minutes of filtration. C) Image after 12 minutes of filtration.

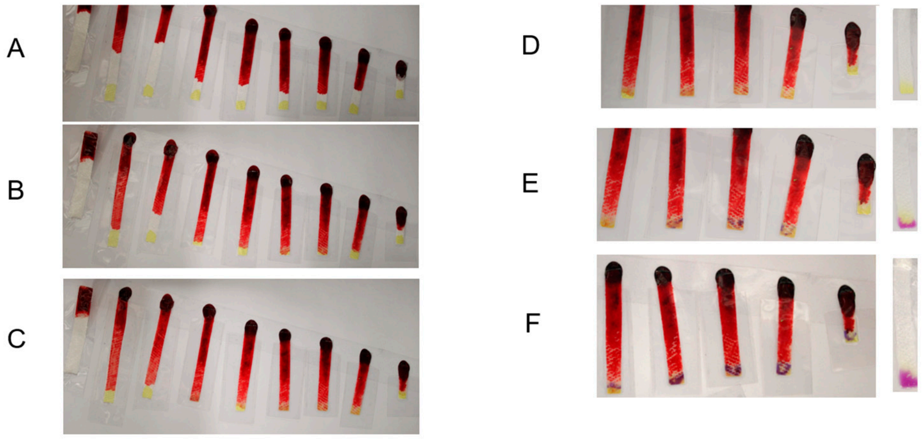

Supplemental Figure S4: Original photographs used for Figure 1E. A) Image after 15 minutes of filtration. B) Image after 30 minutes of filtration. C) Image after 60 minutes of filtration. D) The test strips were then immediately placed on a 40 °C hot plate for the cell-free reaction, at which point the control strip was also hydrated and put on the hot plate. E) Image after 75 minutes, where the final 15 minutes were while the test was on the 40 °C hot plate. F) Image after 85 minutes, where the final 25 minutes were while the test was on the 40 °C hot plate.

Supplemental Figure S5: The equation for standard deviation [2], where *s* is the sample standard deviation, *n* is the number of replicates, x_i is the measurement of a single replicate, and $\bar{x}$ is the average of the replicates.

$$s = \sqrt{\frac{\sum_{i=1}^{n}(x_i - \bar{x})^2}{(n-1)}}$$

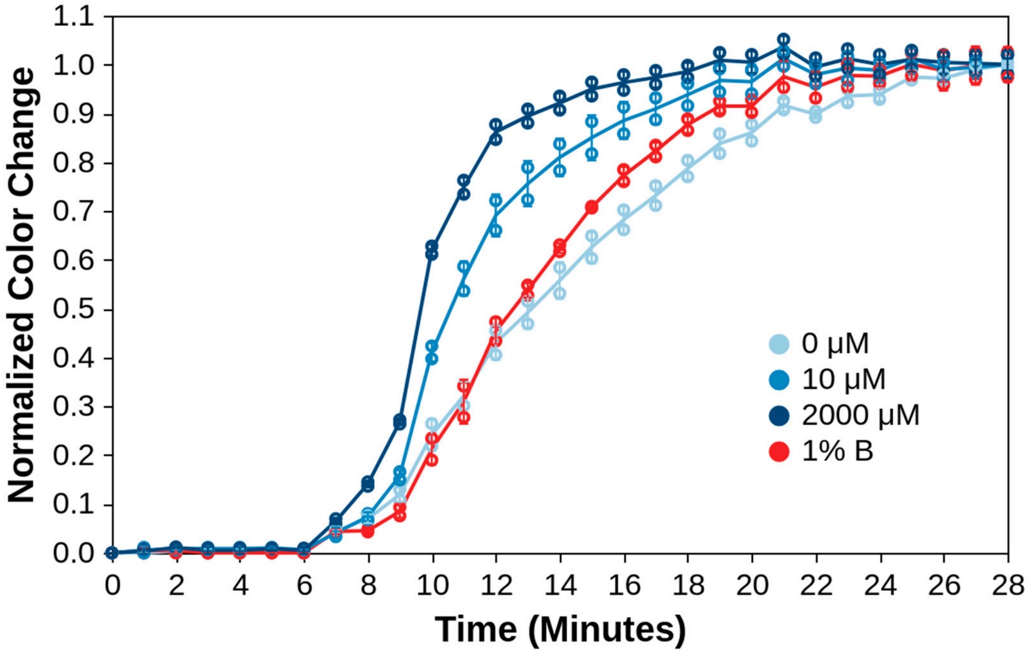

Supplemental Figure S6: Glutamine sensor response plot including individual data points. The plot was obtained with quantitative analysis of sensing reaction photographs for selected conditions from Figure 2C. The average color for each circular reaction paper at each time point was quantified in CIELAB color space and normalized to the starting and ending values within each row. For each time point, the average of replicates for each condition was plotted with interpolating lines and individual data points are shown. The standard deviation for each set of duplicate reactions is displayed as error bars. The labels with concentrations, in μM, indicate the glutamine concentrations in aqueous standards. The 1% B label indicates a 1% blood sample diluted in water.

Supplemental Table S1: Cost estimate for a one replicate of a CFPS blood filtration test

Test Component	Function	Unit Cost	Units Per Test	Cost Per Test
Paper Towel[a]	Blood Cell Filtration	$ 0.000215/in^2	0.5 in^2	$ 0.00011
Transparent Tape[b]	Lamination	$ 0.00313/in^2	2.0 in^2	$ 0.0062
CFPS Reagents[c]	Biosensing Reaction	$ 0.0030/µL	15 µL	$ 0.045
BSA[d]	Paper Pretreatment	$ 0.0029/mg	14 mg	$ 0.041
			**Total**	**$ 0.092 per test**

	Vendor	Product Details
a	Georgia-Pacific, Atlanta, GA	Item 89460
b	J.V. Converting Company, Inc., Fairless Fields, PA, USA	JVCC BOOK-20CC
c	Prepared in house [91]	
d	Millpore Sigma, Burlington, MA	A3294-1KG

Note: MICROSAFE pricing is not published in this work.

References

1. Vitello, D.J.; Ripper, R.M.; Fettiplace, M.R.; Weinberg, G.L.; Vitello, J.M. Blood density is nearly equal to water density: A validation study of the gravimetric method of measuring intraoperative blood loss. J. Vet. Med. **2015**, 2015, 152730.
2. Ramsey, F.; Schafer, D. The Statistical Sleuth: A Course in Methods of Data Analysis; Cengage Learning: Boston, MA, USA, 2012.
